# Factors that impact the patellofemoral contact stress in the TKA: a review

**DOI:** 10.1186/s42836-023-00197-0

**Published:** 2023-08-05

**Authors:** Zhenguo Yu, Hong Cai, Zhongjun Liu

**Affiliations:** 1 Department of Orthopedics II, Beijing Rehabilitation Hospital, Capital Medical University, Xixiazhuang, Shijingshan District, Beijing, 100144 China; 2 Department of Orthopedics, Peking University Third Hospital, No.49 North Garden Road, Haidian District, Beijing, 100191 China

**Keywords:** Total knee arthroplasty, Patellofemoral stress, Contact area, Biomechanics, Anterior knee pain

## Abstract

Abnormal retro patellar stress is believed to contribute to patellofemoral complications after total knee arthroplasty (TKA), but the causal link between TKA and patellofemoral contact stress remains unclear. By reviewing the relevant studies, we found that both TKA implantation and additional patellar resurfacing increase retro patellar pressure. The rotation and size of the femoral component, thickness and position of the patellar component, installation of the tibial component, prosthesis design and soft tissue balance further influence patellofemoral stress. Specific measures can be applied to reduce stress, including the installation of the femoral prosthesis with an appropriate external rotation angle, placing the tibial component at a more posterior position and the patellar button at a more medial position, avoiding over-sized femoral and patellar components, selecting posterior-stabilized design rather than cruciate-retaining design, using gender-specific prosthesis or mobile-bearing TKA system, and releasing the lateral retinaculum or performing partial lateral facetectomy. Despite these measures, the principle of individualization should be followed to optimize the patellofemoral biomechanics.

## Introduction

Total knee arthroplasty (TKA) is becoming an increasingly common procedure for severe knee diseases. Although both the surgical technique and the patient satisfaction rate have been improving in recent decades, some patients still developed patellofemoral complications following TKA, such as persisting anterior knee pain, patellar subluxation or dislocation, patellar component wear or loosening, and even patella fracture [1–3]. The incidence of anterior knee pain is up to 30% [4], resulting from functional and mechanical causes, among others. The former is related to inter- and intramuscular coordination, which can be attributed to preoperative osteoarthritis; the latter concerns increased patellar instability and retro patellar pressure, such as offset errors, over-sizing, rotational errors of the femoral and tibial components, patellar maltracking, patella baja and aseptic loosening, which usually could be radiologically identified [5]. To date, abnormal patellofemoral stress is considered to be one of the principal causes of the above complications [1, 4, 6, 7]. Overloaded patellofemoral joint disrupts tissue homeostasis, including bone and soft tissue homeostasis, and contributes to the perception of anterior knee pain [8]. However, the exact effect of TKA on the force and pressure of the patellofemoral joint remains unclear.

Patellofemoral contact force, area, and pressure are the biomechanical parameters of the patellofemoral joint, and the pressure is defined as the patellofemoral reaction force divided by the contact area between the patella and the surface of the trochlea [9]. Previous studies did not examine exactly the same parameters, most of them mainly measuring the maximum pressure [10–15], with a few studies looking at only the contact force [7, 16, 17] and others examining the force and pressure [9, 18]. In theory, for TKA without patella resurfacing, the measurement of patellofemoral pressure is more valuable than that of the contact force because the former reflects the uneven stress distribution on the articular surface. The minimum bone stress-pain threshold of 271 kPa also adopts the concept of pressure [19], which serves as an effective reference when searching the causes of anterior knee pain with appropriate means such as finite element analysis. For TKA with patellar replacement, however, it might be more meaningful to investigate the patellofemoral force, since the uneven stress between the femoral and patellar components would be transmitted and dispersed to the prosthesis-bone interface by the patellar component.

We reviewed the consistently reported factors in TKA affecting the patellofemoral contact force, area, and pressure, with the aim of providing information for individualized osteotomy and prosthesis installation in TKA and for the diagnosis and treatment of patellofemoral joint complications following TKA.

### Patellofemoral biomechanics

**Fig. 1:**
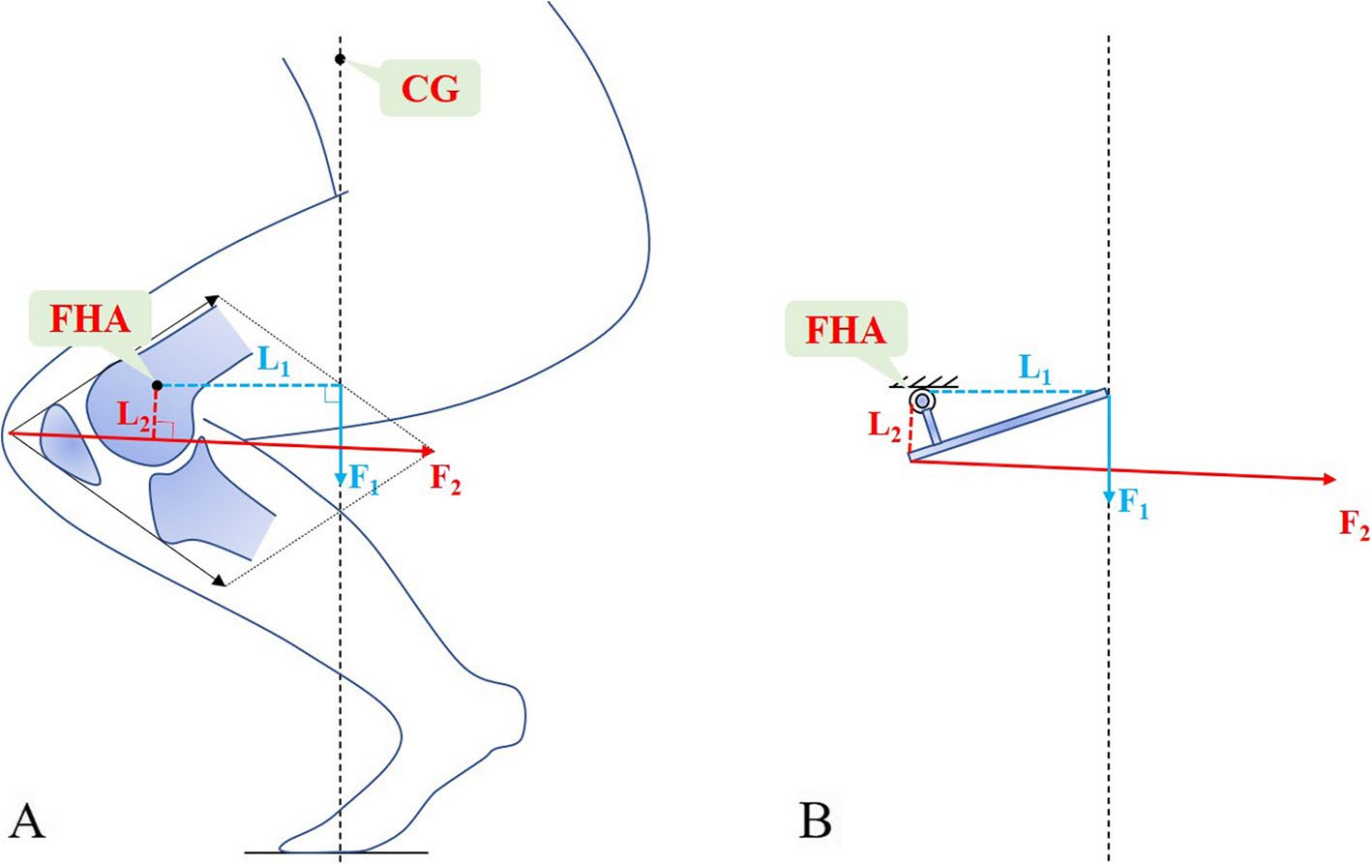
Diagram of the arm and moment of a patellofemoral joint under weight-bearing conditions. **A** FHA = finite helical axis, CG = the body’s center of gravity; F_1_ = body gravity, L_1_ = moment arm of F_1_; F_2_ = the resultant of the quadriceps and patellar tendon forces (i.e., the patellofemoral reaction force), L_2_ = moment arm of F_2_. **B** When balanced, the moment of F_1_ is equal to that of F_2_ (F_1_ × L_1_ = F_2_ × L_2_), simplified as the mode of the lever

As the largest sesamoid bone, the patella functions to force the extensor muscles away from the rotational center of the knee, so as to increase the lever arm of the quadriceps, rendering knee extension more effective [20, 21] (Fig. 1). To put it differently, the presence of patella allows the quadriceps to produce less tension to extend the knee. As to knee flexion, although the patella does not significantly affect the post-femoral muscle group, it reduces the direct friction between the quadriceps and femoral condyles and thereby stabilizes the knee. The longitudinal patellar crest divides the articular surface of the patella into an internal and an external part, and its overall shape fits the trochlear groove. It articulates with the femoral trochlear groove via its vertical ridge that divides the patella into a medial and a lateral facet [20].

The patella engages with the trochlear groove at an approximately 30° knee flexion [22]. Patellofemoral joint contact stress varies constantly throughout the entire range of motion [23]. Lee et al. [24] reported that, physiologically, when the quadriceps tendon tension was at 200 N, the average patellofemoral contact area was 189 ± 83 (mean ± standard deviation), 231 ± 80, 232 ± 80, and 191 ± 51 mm^2^ at 30°, 60°, 90°, and 120°, respectively, while the average pressure was 0.75 ± 0.18, 0.70 ± 0.17, 0.72 ± 0.16, and 0.79 ± 0.24 MPa at the aforementioned four flexion angles, respectively. Regardless of the various values under different test conditions, the changing tendency of both patellofemoral contact area and pressure were comparable across studies [13, 25]. At 90° of flexion, the upper part of the patellar facet articulates with the lower part of the femoral trochlea, and the patella is only in contact with the bilateral femoral condyles at more than 120° of flexion [26]. During normal knee flexion, the patellofemoral contact area gradually moves from the distal to the proximal part on the patella side [23], and the contact area reaches its maximum at 60°–90° of flexion [13, 27]. After 90°, the patella contacts the femoral condyles after breaking away from the trochlear groove, reducing the contact area accordingly [27]. In addition, the decreasing angle between the quadricep tendon and patellar tendon on the sagittal plane leads to an increasing resultant patellofemoral force [28], which is significantly lower at the maximum knee flexion [21].

The quadriceps angle (Q-angle) increases the outward tension applied to the patella, resulting in greater stress on the lateral patellofemoral joint than on the medial facet [25]. In addition, King et al. [1] reported that the normal lateral-to-medial ratios of maximal patellofemoral force and peak pressure reached 1.6 and 1.8, respectively. With the flexion angle increasing, the Q-angle becomes smaller due to the medial rotation of the tibia on the femur, contrary to the so-called screw-home mechanism [23], which affects the distribution of patellofemoral stress.

Rollback of the femoral condyles on the tibial plateau with knee flexion is one of the characteristic features of normal knee kinematics, and is largely attributed to the increasing tension of the posterior cruciate ligament (PCL) during flexion [28]. The range of normal femoral rollback has been reported to be approximately 10–16 mm [28]. With rollback increasing, the rotational center of the tibiofemoral and patellofemoral joint shifts posteriorly, and the patellar moment arm becomes lengthened. A longer patellar moment arm improves the efficiency of the extensor mechanism, which is considered to be one of the causes of significantly decreased patellofemoral force at maximum knee flexion [21]. At deep flexion, however, the reduced aptitude in the patellofemoral contact area exceeded that in the retro patellar force, and, as a result, the patellofemoral pressure continued to increase even in the late stage of the knee flexion [27].

### Influencing factors in TKA on the patellofemoral contact stress

TKA reduces the patellofemoral contact area and elevates the pressure, compared with the normal knee, resulting from the lowered congruence between the femoral and patellar components after TKA [1, 7, 13, 29, 30]. This effect is more prominent following TKA with patellar resurfacing [24, 25, 29]. Similar to the physiological condition of the knee joint, both medial and lateral retro patellar pressures increase with flexion, and the lateral pressure was significantly higher than the medial pressure [16]. After 60° of flexion, the lateral contact pressure exceeded 40 MPa [31]. Moreover, the patellofemoral stress would be further affected by certain factors, such as rotation and size of the femoral component, thickness and position of the patellar component, installation of the tibial component, prosthesis design, and soft tissue balance. Accordingly, optimizing the design and surgical alignment of TKA components to restore physiological patellofemoral biomechanics might prevent those complications [32].

#### Rotation and size of the femoral component

**Fig. 2:**
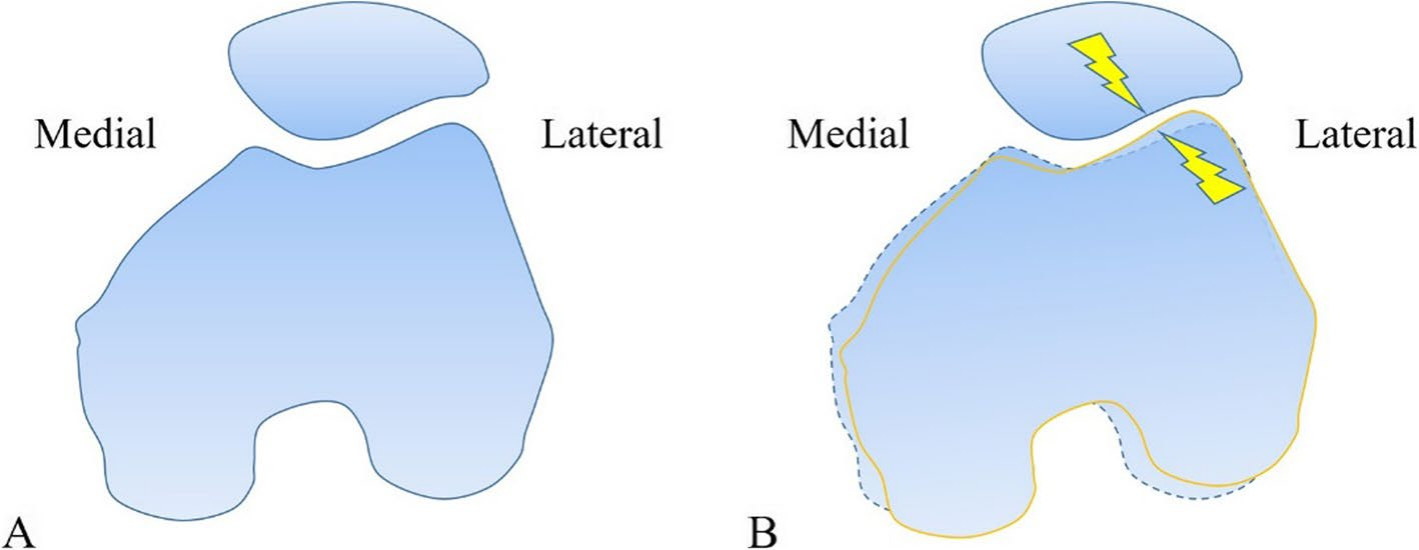
Effect of rotation of the femoral component on the patellofemoral stress. **A** A neutral or external rotation of the femoral component produces a lower stress especially on the lateral part of patellofemoral joint. **B** The center of the contact force shifts laterally with the lateral pressure increasing due to the internal malrotation of the femoral component

Malrotation of the femoral component could contribute to anterior knee pain and accelerate the wear of the patellar component [12]. Some authors even believe it is the most common cause of patellofemoral complications after TKA [5, 33]. This is because that malrotation of the femoral component leads to patellar maltracking and subsequently an abnormal increase in the patellofemoral stress [2, 9, 34, 35]. The patellofemoral pressure is minimal in the neutral configuration, with the contact area being maximal. Malrotation of the femoral component, either internally or externally, alters the patellofemoral contact location, and thereby progressively increases the pressure when the contact area reduces. Specifically, the center of the contact force shifts medially with the medial pressure increasing due to the external malrotation of the femoral component, whereas it moves laterally with an increased lateral pressure resulting from an excessive internal rotation [9] (Fig. 2). This pattern was further confirmed by Louis et al. in a study on femoral malrotation after diaphyseal fracture, resulting in higher patellofemoral stress [10].

Although higher medial stress is induced by external rotation of the femoral component, easing the lateral force benefits patients [28], which might be attributed to a smaller Q-angle. Given a greater stress on the lateral patellofemoral joint compared with the medial facet, an additional increase in lateral stress should be avoided. Thus, for the sake of reducing the maximal retro patellar pressure, Fuchs et al. [13] and Woiczinski et al. [35] both supported a neutral or external rotation of the femoral component. In addition, by using a posterior cruciate-retaining (CR) prosthesis system, Steinbrück et al. [12] recommended a 3°–6° external rotation of the femoral component to the trans-epicondylar axis (TEA) on the premise of ensuring adequate soft tissue balance, which depended on the first step of distal femoral bone cut.

Of note, for the TKA with the patella resurfacing, although the average retro patellar pressure reported in some studies was not up to 20 MPa, malrotation of the femoral component aggravated the malalignment between the patellar button and trochlear groove prosthesis, with the pressure on certain contact points even exceeding the yield stress of ultra-high molecular weight polyethylene (UHMWPE), which exacerbated the wear of the patellar component [9]. Moreover, the rotation of the femoral component has shown a more conspicuous effect on the patellofemoral stress in the range from 45° of flexion to extension [9], which is closely related to daily activities. Therefore, additional attention should be paid to the rotational angles of distal femoral osteotomy, especially for patients who potentially may develop anterior knee pain.

In TKA, the osteotomy amount of femoral condyles and the size of the femoral prosthesis affect the patellofemoral stress as well. Inadequate cutting of the anterior femur (i.e., overhang) or upsized femoral component causes overfilling of the patellofemoral joint, increasing the tension within the patellar tendon and the retro patellar contact force [5, 17, 36]. With the posterior-stabilized (PS) prosthesis, upsizing the femoral component is sometimes necessary to compensate for the increased flexion gap resulting from the resection of the PCL, which is expected to increase the patellofemoral force [17]. In contrast, excessive anterior resection at the distal femur (i.e., notching) and undersized femoral component led to an extension lag, weakness, and patellar instabilities, such as a patellar subluxation or dislocation, due to the smaller moment arm of the quadriceps, despite lower patellofemoral stress. Even with an appropriately-sized femoral prosthesis, its overall shift, either forward or backward, might influence the patellofemoral force as the flexion deficit occurs [5]. Therefore, upon the selection of an appropriately-sized femoral prosthesis, avoiding inadequate cutting of the anterior femur is necessary.

#### Thickness and location of the patellar component

A common issue in TKA, namely, the decision on whether to resurface the patella, remains controversial [13, 25]. Xu et al. [25] conducted a cadaver study and revealed that, in TKA plus patella resurfacing, the patellofemoral pressure increased 9.4- to 29.2-fold compared with the TKA with native patella retained, owing to the markedly decreased contact area after resurfacing of the patella. The higher pressure became substantially greater than the yield stress of UHMWPE. However, two meta-analyses have concluded that there was no difference in the anterior knee pain rate between resurfacing and non-resurfacing groups [37, 38]. Some surgeons resurface the patella routinely when performing TKA [39], while others advocate the preservation of the native patella if there is no high-grade cartilage damage from a biomechanical perspective [30, 40].

For TKA with patella resurfacing, the thickness of the patellar button is the focus of attention. Similar to the impact of femoral component size on patellofemoral stress, a thicker patellar button is expected to lead to patellofemoral overfilling, flexion deficit, and higher patellar tendon tension and retro patellar force. On the other hand, excessive osteotomy is often needed to obtain a smaller overall patellar thickness. It effectively reduces the patellofemoral force, but puts the patella at a greater risk of stress fracture, with increased postoperative complications such as extension weakness and patellar instability [29, 41]. Tanikawa et al. [42] performed a cadaver study with the Triathlon TKA system (Stryker, Kalamazoo, MI, USA) and showed that a 2-mm increase or decrease in patellar thickness resulted in an approximately 20% increase or decrease in the patellofemoral pressure, suggesting surgeons avoid increasing patellar thickness. Moreover, Hsu et al. [29] believed that either a thicker or a thinner patella deteriorated the alignment of the patellofemoral joint and resulted in a smaller contact area than normal-thickness patella. Therefore, surgically, with patellar resurfacing during TKA, effort should be made to restore the original patellar thickness. It is commonly believed that restoration of the original patellar thickness is most desirable [41, 43]. Considering that patients undergoing TKA tend to have a worn or dysplastic patella, and that the size of the femoral component and tension of the retinaculum also influence the reconstruction of total patellar thickness, normal patellofemoral biomechanics might not be able to be re-established if the thickness of the patellar button is determined based on the original patellar thickness measured intraoperatively. Quite on the contrary, a more comprehensive approach accommodating multiple factors should be taken.

The geometry of the patellar facet varies with populations. In an in vivo study on TKA with patellar resurfacing performed on 129 knees, Assi et al. [20] found that 89% of the center of the vertical ridge lay superiorly and medially with reference to the center of the patellar cut. Although no significant difference was noted in the total patellar force when the patellar component shifted [28], its lateralization increased the force on the lateral facet [44]. Another benefit of medialization of the patellar component is decreased lateral shear force on the patella [45]. Therefore, in order not to increase (or even offset partially in theory) the original greater lateral stress, and to restore the patellofemoral biomechanics, it is usually recommended that the patellar button be placed in a medial position relative to the center of the patella. This notion has been accepted by most surgeons [28, 45, 46]. Excessive medialization of the patellar component, however, causes a higher stress on the medial patellofemoral joint. Hence, a modest medialization on the order of 2.5 mm has been empirically proposed [47]. Alternatively, the patellar button can also be placed on the medial two-thirds of the patella, though the measurement might be challenging [45]. Meanwhile, choosing an appropriately larger patella prosthesis can avoid excessive patellar tilt [48], which is a vital contributor to the aggravated the uneven retro patellar pressure distribution. Given that the patella in some patients has a more lateral vertical ridge, Assi et al. [20] proposed a patient-specific approach that factors in the native patellar morphology, to replicate the position of the anatomical patellar center. Panni et al. [49] reported an increased prevalence of the Wiberg Type III patella, with a large lateral facet and a small, more vertical medial facet, in patients with recurrent dislocation of the patella. For these patients, if a TKA is needed, it may be challenging to determine where the patellar button should be installed.

With patella-retaining TKA, scholars tend to reduce or even cut thin the patella besides removing the peripatellar osteophytes. Similar to the effect of the thickness of the patellar button, this operation contributed to a more friendly retro patellar pressure, in theory, despite the lack of support by relevant studies. Meanwhile, reconstructing a more medial highest point of the native patella seems to reduce the already greater pressure of the lateral patellofemoral joint. However, Senioris et al. [50] noted that the patellar morphology and patellofemoral congruence in TKA with unresurfaced patella were not associated with clinical outcomes, indicating that anterior knee pain was probably caused by mechanisms other than patellofemoral pressure.

#### Location and rotation of the tibial component

Compared with the general location of the tibial component in TKA, its anterior displacement increases the patellofemoral force, while its retrodisplacement unloads the patellofemoral joint [27, 51]. On the one hand, the posterior displacement of the tibial component is equivalent to the forward movement of the tibial tubercle, which relieves the tension within the patellar tendon during flexion and extension of the knee, thus reducing the stress in the patellofemoral joint. On the other hand, as the tibial component shifts backward, so does the rotational center of the knee joint, and the moment arm of the quadriceps increases, thereby enhancing the efficiency of the extensor mechanism. In addition, the aforementioned effect from the anteroposterior position of the tibial prosthesis is particularly evident at an over 90° flexion [27]. Therefore, given the advantage of unloading the patellofemoral joint, a more posterior position of the tibial component is beneficial, especially for patients with deep flexion pain.

As to whether and how the rotation of the tibial component affects patellofemoral stress, no reports are available to date. However, in an in vivo study, Nicoll and Rowley [52] stated that internal rotational error of the tibial component was a major cause of anterior knee pain following TKA, while its external rotational error could be well tolerated. Within this relationship, abnormal stress in the patellofemoral joint might be the mechanistic link between the internal rotation of the tibial component and anterior knee pain.

#### Prosthesis design

**Fig. 3:**
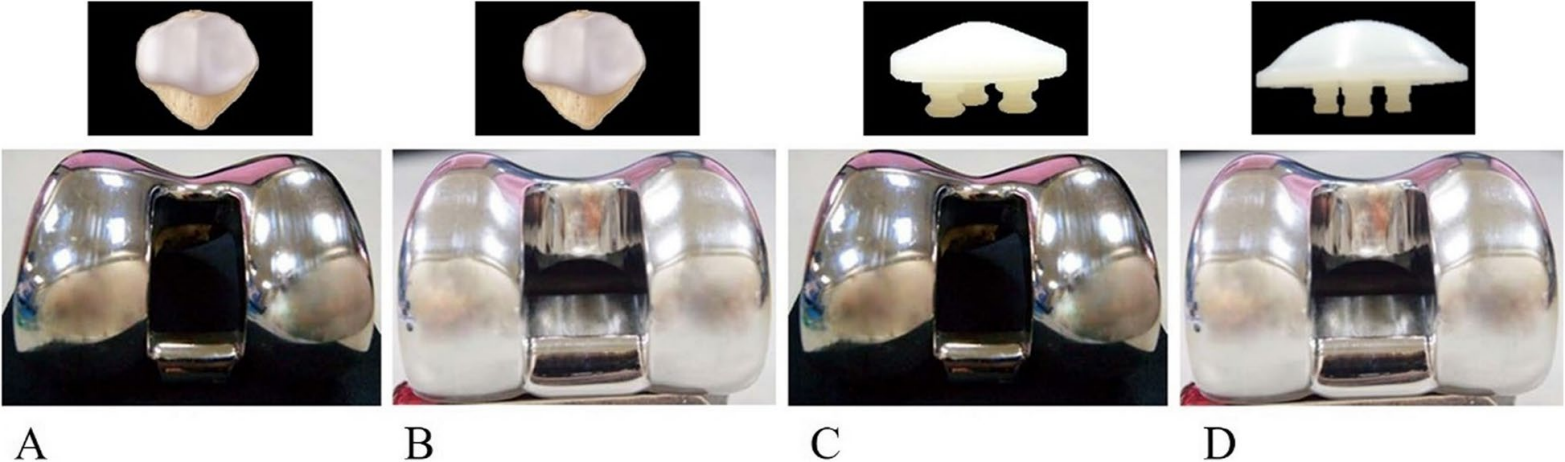
Effect of trochlear groove morphology on the patellofemoral stress. The femoral component with a V-shaped trochlear groove reduced the compressive strain on the unresurfaced patella, but when the patella was resurfaced, a femoral component with a curved dome-shaped design reduced the strain in the remaining patellar bone [11]. **A** V-shaped trochlear groove with unresurfaced patella; **B** dome-shaped trochlear groove with unresurfaced patella; **C** V-shaped trochlear groove with resurfaced patella; **D** dome-shaped trochlear groove with resurfaced patella

To date, a wide array of prostheses have been available for TKA, with different sagittal radii, depths and orientations of the trochlear groove of the femur as well as the geometry of the patellar component surface in the patellofemoral design [28]. The implant design regarding the morphology of the trochlear groove could affect patellofemoral contact stress following TKA [53]. By designing different trochlear grooves based on Genesis II total knee endoprosthesis (Smith & Nephew), Leichtle et al. [54] believed that the original design (imitating native knee with slight lateral elevation) of the trochlear groove had a more extensive patellofemoral contact area than the flat and deeply-recessed one and that the flat profile elevated patellofemoral peak pressures, at low flexion angles, up to approximately 50°. Huang et al. [11] further investigated the effect of trochlear groove morphology on the stress distribution of the patellofemoral joint with the NexGen PS LPS-Flex fixed bearing knee system (Zimmer, Warsaw, IN, USA) with or without patella resurfacing respectively. They found that the femoral component with a V-shaped trochlear groove reduced the compressive strain on the unresurfaced patella, but when resurfacing the patella, a femoral component with a curved dome-shaped design might reduce the strain of the remaining patellar bone (Fig. 3). This might be because the articulating geometry of an anatomical V-shaped design was closer to the morphology of the native patella, while the loading on the rounded articular surface in the dome-shaped design model could be more evenly transferred across the patellar component than in the V-shaped design model and decrease the strain on the remaining patella [11]. Therefore, choosing an appropriate femoral component for different procedures would help reduce patellofemoral complications after TKA. It is noteworthy that the elevation of the lateral margin of the trochlear groove, is essential for providing mediolateral guidance and avoiding patellar instability, and pressure on the lateral patellar facet increased. This indicates that an excessively elevated lateral margin is not conducive to the re-establishment of the patellofemoral biomechanics [54]. In addition, Schindler et al. [55] pointed out that the patellofemoral contact area, which is highly dependent on the congruency of the patellofemoral joint articulation, was significantly greater with the dome-shaped patellar component than for that with a more anatomic design when engaging in three-dimensional movement. On the basis of a follow-up lasting more than 10 years, Karachalios et al. [56] concluded that anatomical, patella-friendly, and constant radius femoral components had an advantage in reducing anterior knee pain and patellar complications, which might be attributed to the more superior patellofemoral biomechanics.

The selection of tibiofemoral implants also influences the patellofemoral biomechanics. A few studies believed that after implantation of the PS prosthesis, average patellofemoral contact force and pressure were significantly lower in comparison to the posterior CR design [7, 14, 21]. This is because PS TKA allows for the rollback of femoral condyles so that it lowers the patellofemoral strain to some extent [7, 14], while the distal femur with CR design even has a reverse roll forward which increases the retro patellar pressure [57]. However, a meta-analysis found no difference in postoperative anterior knee pain between PS and CR TKA [58], suggesting that patellofemoral contact force is not the sole cause of postoperative knee pain. A cadaver study by Johanson et al. [18] revealed that, at 0°–130° knee flexion, with a gender-specific femoral component, which has a thinner anterior flange, the patellofemoral peak pressure was smaller as compared with conventional prosthesis. In addition, gender-specific components also contributed to decreased ratios of lateral-to-medial patellofemoral forces or pressures, and helped to partially relieve the higher pressure on the lateral patellar facet induced by TKA [1]. Nonetheless, Kawahara et al. [17] found no difference in the mean patellofemoral contact force between the gender-specific and standard components of the same size at deep flexion.

In TKA, the mobile-bearing design showed evidently lower patellofemoral contact stress than its fixed-bearing counterpart [59, 60]. This was attributed to the additional advantage of the self-aligning feature of the mobile-bearing TKA system [60], and the potential ability of the mobile-bearing design to imitate the asymmetrical rollback of femoral condyles [59], which reduces the lateral retro patellar pressure. Increased pressure on the medial facet is induced theoretically at the same time, but given that lateral pressure tends to be higher under common conditions [61], the mobile-bearing TKA still presents some advantages over the fixed one in terms of relieved overall patellofemoral contact stress. In addition, during knee flexion, the restoration of the femoral rollback improved the efficiency of the extensor mechanism, further reducing patellofemoral stress. D’Lima et al. [28] reported that a consistent reduction of up to 7% in patellofemoral forces was seen with progressive magnitudes of femoral rollback of 10 mm, and, as expected, femoral roll forward increased patellofemoral force.

#### Soft tissue balance

Intraoperative soft tissue balance also influences retro patellar stress [16]. For instance, the patellofemoral contact pressure could be elevated by the iatrogenic injury to the posterior soft tissue stabilizers (PCL, posteromedial and posterolateral corner) due to the posterior subluxation of the tibia, especially during flexion [5]. To take another example, any vastus medialis imbalance makes the patella susceptible to lateral subluxation and results in increased lateral condylar contact pressure [26].

For patients with patellar instability, the lateral retinaculum release is a common surgical technique. It has been proven that lateral retinaculum release was able to decrease the retro patellar contact force and pressure [16, 62, 63], with an additional elevation of the medial facet [61]. Thus, the ratio of lateral-to-medial patellofemoral stress decreases, which resembles the effect of the gender-specific component [1]. As Zha et al. [63] suggested, routine lateral retinacular release in TKA could reduce the morbidity of anterior knee pain without increasing lateral retinacular release-related complications. Nevertheless, in a cadaver study with an average age of 82 years, Peretz et al. [61] observed that lateral peak pressure decreased after lateral release when using a standard prosthesis, but in the gender-specific component, both lateral and medial peak pressures increased after lateral release. Therefore, a combination of lateral retinacular release and gender-specific components is not conducive to optimizing patellofemoral stress.

In addition, lateral facetectomy can reduce patellofemoral contact pressure in non-patella-resurfacing TKA by improving the alignment between the native patella and trochlear groove prosthesis [64]. Lakstein et al. [65] found improved postoperative anterior knee pain after partially resecting the lateral facet, which could be applied as an alternative to lateral release for managing patellar maltracking and stress abnormality.

Despite numerous approaches available to reduce patellofemoral stress, not all are applicable to specific individuals, and the appropriate strategies should be used. For instance, for patients with dysplasia of the distal femur (excessive internal rotation angle), increasing the external rotation angle properly during osteotomy might be a better choice to improve patellofemoral biomechanics. One example is that, for a patient with preoperative patellar maltracking, the surgeon could balance the soft tissues around the patella (such as releasing the lateral retraculum) to optimize patellar movement patterns and decrease retro patellar stress. Additionally, it is necessary to combine two or more methods to achieve the goal at times. At present, however, literature remains scanty on individualized treatments, and further researches are urgently needed.

## Conclusion

Both TKA implantation and additional patellar resurfacing significantly influence retro patellar pressure. The rotation and size of the femoral component, thickness and position of the patellar component, installation of the tibial component, prosthesis design, and soft tissue balance further affect patellofemoral stress. Specific measures can be applied to reduce stress, including the installation of the femoral prosthesis with an appropriate external rotation angle (3°–6° external rotation to the TEA), placing the tibial component at a more posterior position and the patellar button at a more medial position, avoiding over-sized femoral and patellar components, selecting PS design rather than CR design, using gender-specific prosthesis or mobile-bearing TKA system, and releasing the lateral retinaculum or performing partial lateral facetectomy. Despite these alternatives, an individualization principle should be followed to optimize the patellofemoral biomechanics. Additionally, most of the previous studies on patellofemoral stress were in vitro studies or finite element model analysis, lacking direct evidence of the relationship between abnormal retro patellar stress and postoperative complications. Therefore, it will be valuable to investigate the patellofemoral stress during TKA with subsequent follow-up of patellofemoral joint complications such as anterior knee pain after TKA, which will contribute to the improvement of its clinical results.

## Data Availability

Not applicable.

## Abbreviation

TKA: Total knee arthroplasty
Q-angle: Quadriceps angle
PCL: Posterior cruciate ligament
TEA: Trans-epicondylar axis
UHMWPE: Ultra-high molecular weight polyethylene
PS: Posterior-stabilized
CR: Cruciate-retaining
